# Supplementary material for: Drug repurposing for aging research using model organisms
Source: Aging Cell. 2017 Jun 16;16(5):1006–15. doi: 10.1111/acel.12626 (PMC5595691; doi:10.1111/acel.12626)
Supplement: Supplementary file 7 — Data S1 Zip‐Archive of all report cards. [file ACEL-16-1006-s007.zip › RC_DFM.pdf]

DFM

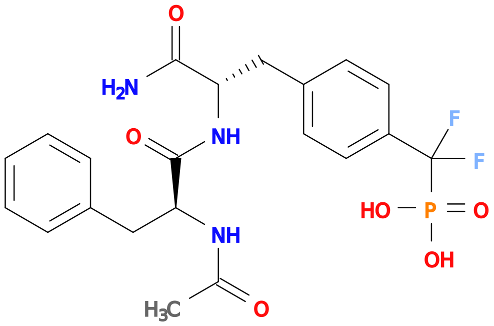

Database identifiers

ChEMBLCompound	CHEMBL196526
DrugBank	DB07651
CHEBI	47379

Ranking

	Rank	Score
Drosophila	586/697	0.168
C. elegans	NA	NA

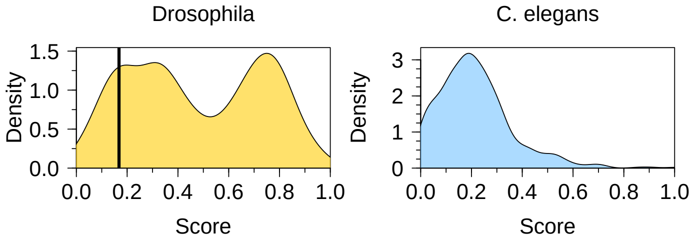

	Ageing implication	Domain conservation	Binding site conservation	Binding affinity	Bioavailability	Lipinski	Promiscuity	Purchasability	Drug approval	**Total**
Drosophila	0.203	0.88	1.0	0.583	(0.9)	0.0	-0.0	0.0	0.075	0.168
C. elegans	NA	NA	NA	NA	NA	NA	NA	NA	NA	NA

Names

- **N-acetyl-L-phenylalanyl-4-[difluoro(phosphono)methyl]-L-phenylalaninamide**
- (4-(S)-2-((S)-2-Acetylamino-3-phenyl-propionylamino)-2-carbamoyl-ethyl-phenyl-difluoro-methyl)-phosphonic acid

Roles

EC 3.1.3.48 (protein-tyrosine-phosphatase) inhibitor

Status

Approved drug (according to ChEMBL)	No
Classification (according to DrugBank)	experimental
Number of Rule of 5 violations	0
Binding affinity to original target in log units (RF-Score prediction)	5.33
Burns *C. elegans* bioavailability prediction	-2.64

Compound Target Characteristics

Tyrosine-protein phosphatase non-receptor type 1

Best gene implication in ageing for this target family came from gene P18031 via mapping the annotation from Ensembl ENSG00000196396 via mapping the annotation from EntrezGene 5770 via mapping the annotation from GenAgeHuman 0033 annotated in GenAge release 17.

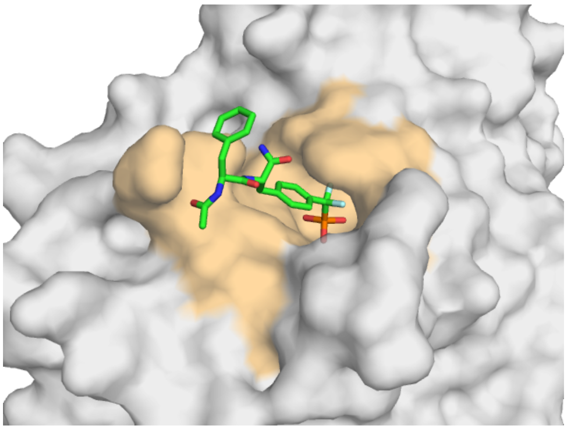

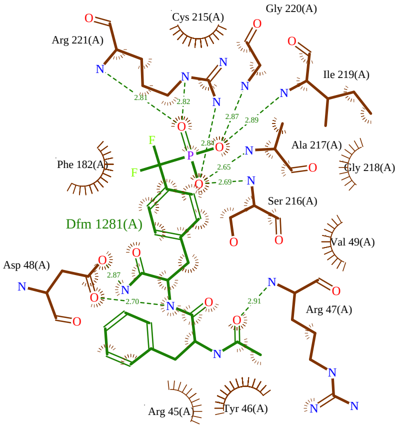

protein	amino acids contacts (binding site)
PDB:2cmc:chainA:P18031	R Y R D V D F C S A G I G R Q
sp:P18031:PTN1_HUMAN	R Y R D V D F C S A G I G R Q
tr:A8K3M3:A8K3M3_HUMAN	R Y R D V D F C S A G I G R Q
sp:P20417:PTN1_RAT	R Y R D V D F C S A G I G R Q
tr:Q3TB93:Q3TB93_MOUSE	R Y R D V D F C S A G I G R Q
tr:Q3TZW9:Q3TZW9_MOUSE	R Y R D V D F C S A G I G R Q
sp:P35821:PTN1_MOUSE	R Y R D V D F C S A G I G R Q
sp:Q9W0G1:PTP61_DROME	R Y R D V D F C S A G I G R Q
tr:D3DMI1:D3DMI1_DROME	R Y R D V D F C S A G I G R Q
tr:I0B1P6:I0B1P6_DROME	R Y R D V D F C S A G I G R Q

	whole protein		domain-based		contact-based	
protein	ident	simil	ident	simil	ident	simil
PDB:2cmc:chainA:P18031	0.98	0.98	1.0	1.0	1.0	1.0
sp:P18031:PTN1_HUMAN	1.0	1.0	1.0	1.0	1.0	1.0
tr:A8K3M3:A8K3M3_HUMAN	1.0	1.0	1.0	1.0	1.0	1.0
sp:P20417:PTN1_RAT	0.84	0.94	0.96	0.99	1.0	1.0
tr:Q3TB93:Q3TB93_MOUSE	0.7	0.73	0.95	0.99	1.0	1.0
tr:Q3TZW9:Q3TZW9_MOUSE	0.83	0.94	0.95	0.99	1.0	1.0
sp:P35821:PTN1_MOUSE	0.83	0.94	0.95	0.99	1.0	1.0
sp:Q9W0G1:PTP61_DROME	0.3	0.59	0.49	0.8	1.0	1.0
tr:D3DMI1:D3DMI1_DROME	0.3	0.59	0.49	0.8	1.0	1.0
tr:I0B1P6:I0B1P6_DROME	0.3	0.59	0.49	0.8	1.0	1.0

Ptp61F (UniProt:Q9W0G1) annotation

Function: Non-receptor protein tyrosine phosphatase required for maintaining Dock in its non-phosphorylated state. (PubMed:12014990, PubMed:8463208).
Subcellular location: Isoform A: Cytoplasm. Endomembrane system. Note=Associates with the membranes of the reticular network and the mitochondria.
Subcellular location: Isoform B: Nucleus.
Tissue specificity: Expressed during oogenesis and embryogenesis. Isoform A and isoform B are expressed in distinct patterns. Isoform B is expressed in the mesoderm and neuroblast layer during germband extension and later in the gut epithelia. Isoform A accumulates in 16 segmentally repeated stripes in the ectoderm during germband extension. These stripes are flanked by, and adjacent to, the domains of engrailed and wingless gene expression in the anterior/posterior axis. In

stage 10 embryos, isoform A colocalizes with the area lateral to the denticle belts that will give rise to naked cuticle. Isoform A is also expressed later in embryogenesis in the central nervous system. (PubMed:9256342).

(Information from UniProt)
